# MapSentinel: Can the Knowledge of Space Use Improve Indoor Tracking Further?

**DOI:** 10.3390/s16040472

**Published:** 2016-04-02

**Authors:** Ruoxi Jia, Ming Jin, Han Zou, Yigitcan Yesilata, Lihua Xie, Costas Spanos

**Affiliations:** 1 Department of Electrical Engineering and Computer Sciences, University of California, Berkeley, CA 94720, USA; jinming@berkeley.edu (M.J.); spanos@berkeley.edu (C.S.); 2 School of Electrical and Electronics Engineering, Nanyang Technological University, Singapore 639798, Singapore; zouhan@ntu.edu.sg (H.Z.); elhxie@ntu.edu.sg (L.X.); 3 Department of Electrical and Electronics Engineering, Middle East Technical University, Ankara 06800, Turkey; yigitcan.yesilata@metu.edu.tr

**Keywords:** indoor tracking systems, non-intrusive, map-aided, WiFi, ultrasonic sensor networks, particle filters

## Abstract

Estimating an occupant’s location is arguably the most fundamental sensing task in smart buildings. The applications for fine-grained, responsive building operations require the location sensing systems to provide location estimates in real time, also known as indoor tracking. Existing indoor tracking systems require occupants to carry specialized devices or install programs on their smartphone to collect inertial sensing data. In this paper, we propose MapSentinel, which performs non-intrusive location sensing based on WiFi access points and ultrasonic sensors. MapSentinel combines the noisy sensor readings with the floormap information to estimate locations. One key observation supporting our work is that occupants exhibit distinctive motion characteristics at different locations on the floormap, e.g., constrained motion along the corridor or in the cubicle zones, and free movement in the open space. While extensive research has been performed on using a floormap as a tool to obtain correct walking trajectories without wall-crossings, there have been few attempts to incorporate the knowledge of space use available from the floormap into the location estimation. This paper argues that the knowledge of space use as an additional information source presents new opportunities for indoor tracking. The fusion of heterogeneous information is theoretically formulated within the Factor Graph framework, and the Context-Augmented Particle Filtering algorithm is developed to efficiently solve real-time walking trajectories. Our evaluation in a large office space shows that the MapSentinel can achieve accuracy improvement of 31.3% compared with the purely WiFi-based tracking system.

## 1. Introduction

The indoor location sensing technology has emerged as an inherent part of the “smart buildings” as it provides great potential for building operation improvement and energy saving. For instance, an on-demand ventilation or lighting control policy must know the usage of the building spaces, which may involve when building occupants enter or exit the building, where they inhabit, what time they occupy the spaces, the duration of occupancy, *etc*. Such applications require the location sensing systems to provide real-time estimate of occupants’ locations, which is also termed “indoor tracking”, in order to realize fine-grained, responsive building operations.

Most indoor tracking systems necessitate each occupant to carry or wear a powered device such as an infrared [1], ultrasonic [2,3,4], or Radio Frequency transceiver [5,6,7]. Even if the transceiver is miniaturized into a convenient form, occupants are not willing or likely to carry it at all times. Another subset of tracking systems alleviate the need for carrying specialized devices by using the inertial sensors on smartphones to perform dead reckoning [8,9,10]. However, specialized programs are required to be installed on smartphones to continuously collect inertial sensing data, and thereby the associated energy issues or occupants’ engagement become the main impediment.

On the contrary, we enable non-intrusive indoor tracking by developing an information fusion system that takes advantage of noisy measurements from various sensors, namely, WiFi access points and ultrasonic sensors. WiFi access points are beneficial for wide spatial coverage while WiFi signals transmitted in the indoor environments suffer from large variations [11]; ultrasonic sensors are able to accurately locate the occupants in their detection zones which are nevertheless limited spatially. Our vision is of occupants carrying some device with WiFi module, which can be smartphones, tablets, wearable devices, *etc.*, in the indoor space where ultrasonic sensors can provide opportunistic calibration of the location estimation. The location sensing system is operating in a passive way, *i.e.*, there is no need for specialized devices or programs for location inference.

In addition to the sensor measurements, another key input for our system is the floormap of the indoor space of interest. Floormap information has been used to refine walking trajectory estimates by eliminating wall-crossings or unfeasible locations [12,13,14]. There has also been efforts to use the floormap to reduce the complexity of the tracking task by properly quantizing the indoor space [15,16,17,18]. In effect, we can also acquire some prior knowledge of occupants’ dynamic motion from the floormap. The indoor space comprises several typical components, such as cubicles, offices, corridors, open areas, *etc.*, where occupants’ motion exhibit distinctive patterns. For example, when located at his/her office or cubicle, the occupant is very likely to keep static; the occupant walking on a particular corridor tends to continue the motion constrained along the corridor, while an occupant in an open space is free to move in any direction. Such information of space use is useful to track occupants’ movement, notwithstanding it is less considered in previous work. Gusenbauer *et al*. [19] exploited different types of movements to improve the tracking model. This was done by introducing an activity recognition algorithm based on accelerometer data to model pedestrians’ steps more reliably. Park [20] proposed incorporating the floormap information by “path compatibility”, where occupants’ motion sequences and motion-related information (e.g., duration and speed) are first estimated based on mobile sensing data, and then localization is achieved via matching occupants’ motion sequences and the hypothetical trajectories provided by the floormap. Kaiser *et al*. [21] proposed a motion model based on the floormap, which weights the possible headings of the pedestrian as a function of the local environment. Our work differs from [19] and [20] in that our work does not rely on the inertial measurements to recognize the motion. Instead, the motion information is extracted from the floormap. We exploit the prior knowledge that the floormap endows us about the occupants’ typical movement and activity, not merely the possible headings at each point of the floormap as in [21]. It is, therefore, the objective of this paper to propose MapSentinel, a non-intrusive location sensing system via information fusion, which combines the various sensor measurements with the floormap information, not only as a sanity check of estimating trajectories but as an input for occupants’ kinematic models.

Our main contributions are as follows:

- We build a non-intrusive location sensing network consisting of modified WiFi access points and ultrasonic calibration stations, which does not require the occupants to install any specialized programs on their smartphones and prevents the energy and occupant engagement issues.
- We propose an information fusion framework for indoor tracking, which theoretically formalizes the fusion of the floormap information and the noisy sensor data using Factor Graph. The Context-Augmented Particle Filtering algorithm is developed to efficiently solve the walking trajectories in real time. The fusion framework can flexibly graft floormap information onto other types of tracking systems, not limited to the WiFi tracking schemes that we will demonstrate in this paper.
- We evaluate our system in a large typical office environment, and our tracking system can achieve significant tracking accuracy improvement over the purely WiFi-based tracking systems.

The rest of this paper expands on each of these contributions. We conclude the paper and discuss the future work in Section 6.

## 2. MapSentinel Architecture

**Figure 1:**
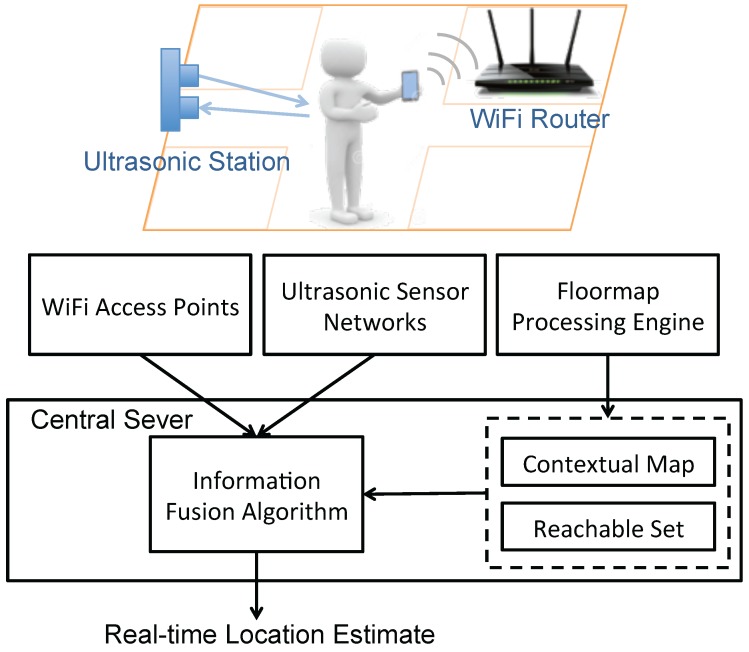
MapSentinel architecture—WiFi APs keep tracking occupants’ locations, and the estimation is periodically refined using the ultrasonic stations deployed in the environment. Furthermore, the sensor measurements and the floormap information are combined via the information fusion algorithm to estimate location in real-time. The floormap processing engine helps transform the floormap to the information accessible to the fusion algorithm.

Figure 1 presents the overall architecture of MapSentinel. There are three key components in MapSentinel: the non-intrusive sensing networks, the floormap processing engine, and the information fusion algorithm. The non-intrusive sensing networks, as the name suggests, generate location-related measurements without the need for computation on the smartphone end. Our sensing networks consist of WiFi access points (APs) and ultrasonic calibration stations, which track locations by relating the WiFi signal strength or the sound time-of-flight to the distance. The floormap processing engine converts the pictorial floormap to the information that can be directly combined with the sensor measurements in the fusion algorithm. The output of the floormap processing engine represents the prior knowledge obtained from the map, and can be computed in the offline phase. We will present the details of the main components of MapSentinel in this section.

### 2.1. WiFi Access Points

IEEE 802.11 (WiFi) is the most commonly used wireless networking technology with widely available infrastructure in large numbers of commercial and residential buildings. Nearly every existing commercial mobile device is WiFi enabled. The common method to utilize WiFi for indoor location sensing is to enable the mobile device to collect WiFi Received Signal Strengths (RSS) of nearby WiFi APs by installing an application on the mobile devices. Our system, on the contrary, leverages WiFi in a non-intrusive manner. Rather than modifying the hardware or software of occupants’ mobile devices, we upgrade the software of the existing commercial WiFi APs to allow them to detect the RSS of each mobile device, while providing basic internet service to occupants as well. The RSS and media access control (MAC) address of each mobile device will be forwarded to the server and the occupant can be identified through the unique MAC address of the mobile device.

### 2.2. Ultrasonic Calibration Stations

**Figure 2:**
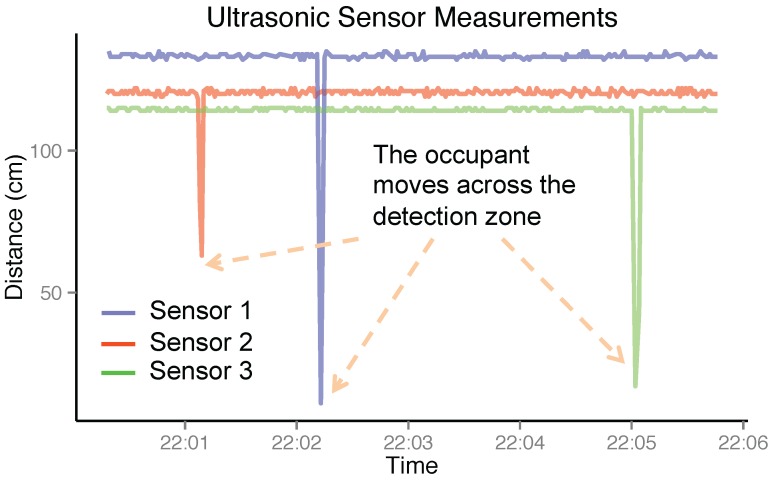
The measurements of ultrasonic stations deployed in the space. When the occupant is within the detection zone of the ultrasonic station, the sensor reading exhibits a smaller value.

Ultrasonic sensors measure the distance to the obstacle in the front to accurately position the object in its detecting range, which works by detecting the time of return, *t*, and the distance is given by:

(1) $$d=\frac{v_{\text{sound}}\times t}{2}$$

where $v_{\text{sound}}\approx 340$ m/s is the velocity of sound in the air. The advantages include centimeter-resolution distance measurements and limited span of detection angles, which make it suitable for online calibration of indoor positioning systems. Figure 2 demonstrates typical traces of the ultrasonic sensor readings when the occupant moves across the detection zones. By properly thresholding the distance measurements, the ultrasonic sensor can be used as an indicator of occupant presence inside its detection zone.

**Figure 3:**
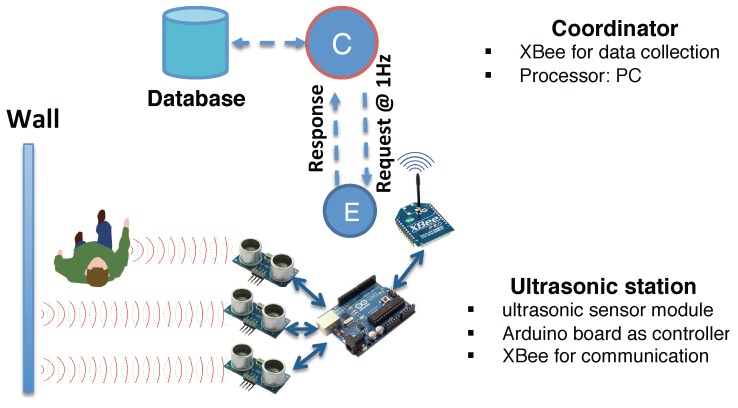
Illustration of the configuration of the ultrasonic calibration station. The coordinator requests measurements at 1 Hz frequency through the IEEE 802.15.4 protocol, and deposits collected data to the local database. The ultrasonic station takes three independent measures from its sensor points to detect occupant presence in the vicinity.

The network consists of deployed ultrasonic stations and data collection center, which communicate with XBee radio modules operating the IEEE 802.15.4 standard, more specifically, the ZigBee protocols, as shown in Figure 3. The radios are low-power and can operate reliably in the indoor space, where the network can be automatically established by the coordinator, in our case, the data collection center. The data center controlled by Arduino enquires about the ultrasonic station for measurements periodically, so that the measurement frequency is 1 Hz, and transfers the data to the computer connected by serial ports. Each ultrasonic station is equipped with three ultrasonic sensors, whose directions are offset by 15${}^{\circ }$. As the measurement range spans 15${}^{\circ }$ for each ultrasound, this covers an area of 45${}^{\circ }$ in the front of the station, which is sufficient for indoor area localization.

### 2.3. Floormap Processing Engine

The indoor space is well structured and typically organized into corridors, open areas, walls, rooms, *etc*. Depending on the occupant’s present location, the motion is constrained by these external factors. For instance, an occupant on a particular corridor has high probability continuing its motion constrained along the corridor—or an occupant walking in the open area is free to move in any direction. Likewise, an occupant in his/her cubicle area is more likely to stay static. Based on different motion capabilities, we categorize the indoor space into several contexts, namely, open space, constrained space and static space. In addition, the floormap processing engine is designed to convert the original floormap into the *contextual floormap* that indicates the context of each point in the original floormap. The details of each component of contextual floormap is provided in Table 1. We use the word “canonical direction” to refer to the direction of constrained space along which the movement has more freedom.

In addition, the occupant motion is also constrained by speed restrictions. Another function of the floormap processing engine is to compute the reachable set containing all the points visited with admissible speed from a given starting point. In the indoor space, the geographical distance between two positions in a floormap does not necessarily equal to the walking distance between them due to the block of walls and other obstacles. Hence, the physical features of the indoor environments would be ignored if the reachable set is confined within a fixed radius centered around the given starting point. The floormap processing engine addresses this problem by converting the floormap to a graph where all the non-barricade nodes connect to their neighboring non-barricade nodes and the barricade nodes do not have connections to any other nodes. In this way, the reachable set of a given node can be computed through finding the nodes within the maximum depth from the root node, which can be efficiently solved by breadth-first search algorithm [22].

## 3. Information Fusion Framework

In this section, we propose an information fusion framework that manages the heterogeneous sensor measurements as well as the floormap and occupants’ context-related motion characteristics to provide an online estimate of occupants’ location. There are two key components in the fusion framework: Context-Dependent Kinematic Models (CDKM) and Probabilistic Sensor Measurement Models (PSMM). CDKM is based on the observation that occupants’ movements exhibit distinctive features in different parts of buildings as described in Section 2.3, and it captures this context-dependency by defining different kinematic models for distinctive contexts. PSMM models each sensor measurement as a probability distribution and multiple sensor data are combined via Bayes’ rule to support the location inference.

### 3.1. Problem Formulation

Consider that the indoor space of interest is composed of *M* contexts, in each of which occupants exhibit a particular sort of kinematic patterns. Denote the context at time *k* as $m_{k}$ where $m_{k}\in \left\{FS,CS_{1},\cdots ,CS_{R},SS\right\}$. The subscript of CS represents the index of the certain direction of constrained space and *R* is the total number of different directions. Let the state $x_{k}=\left(z_{k},m_{k}\right)$ consist of the position and velocity components of the occupant in the Cartesian coordinates $z_{k}=\left(x_{k},y_{k},{\dot{x}}_{k},{\dot{y}}_{k}\right)$ , as well as the context $m_{k}$. If the position is known, the context can be uniquely determined by the contextual floormap. We characterize this correspondence via a function $M:R^{4}\to R$ which assigns a specific context $m_{k}$ for $z_{k}$. The tracking problem can be viewed as a statistical filtering problem where $z_{k}$ is to be estimated based on a set of noisy measurements $y_{1:k}=\left\{y_{1},\cdots ,y_{k}\right\}$ up to time *k*. Specifically, $y_{k}$ is the measurements available at time *k*, and, in our case, it includes measurements from multiple sensors, ${\left\{y_{k}^{n}\right\}}_{n=1}^{N_{s}}$ where $N_{s}$ is total number of sensors deployed in the space of interest. We model the uncertainty about the observations and the states by treating them as random variables and assigning certain probability distribution to each random variable. In this setting, we want to compute the posterior distribution of the state given the measurements up to time *k*, *i.e.*, $p(z_{k}|y_{1:k})$.

**Figure 4:**
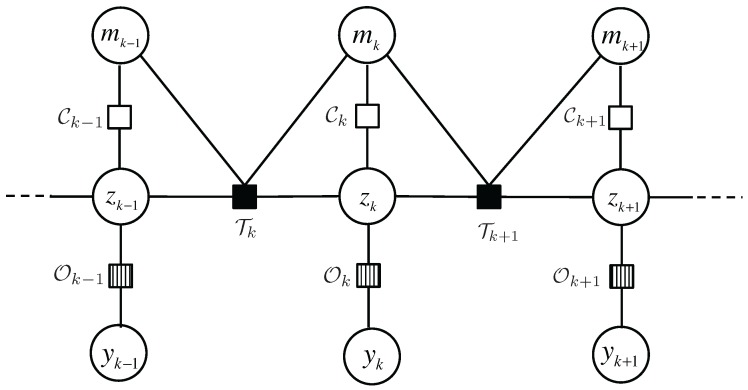
A factor graph model representation of the dependencies among location, velocity, context and observation.

The impact of introducing context as an auxiliary state variable is manifold. Firstly, the transition of contexts $m_{k-1}$ to $m_{k}$ determines the type of motion executed during the time interval (k−1,k]. For instance, if the context remains the same, then the occupant should follow the motion type defined by the two identical contexts; on the contrary, if the context varies during (k−1,k], then the occupant would execute the motion that is defined by neither of the contexts. For simplicity, we will assume a free motion. That is, the position/velocity state at time *k*, $z_{k}$, depends on not only the past state $z_{k-1}$ and $m_{k-1}$, but also the current context $m_{k}$ stochastically. Moreover, there is a deterministic mapping between $z_{k}$ and $m_{k}$ as is specified by the contextual map. In order to facilitate visualization and analysis of the complex dependencies among the variables, we use a factor graph to represent the states, observations and the functions bridging these variables, as illustrated in Figure 4.

A factor graph has two types of nodes, *variable node* for each variable and *function node* for each local function, which are indicated by circles and squares, respectively. The edges in the graph represents the “is an argument of” relation between variables and local functions. For example, the function $T_{k}$ has four arguments, $z_{k}$, $z_{k-1}$, $m_{k-1}$ and $m_{k}$. Three types of local functions are involved in our model:

- $T_{k}\left(z_{k},z_{k-1},m_{k},m_{k-1}\right)=p\left(z_{k}|z_{k-1},m_{k},m_{k-1}\right)$: transition model, or the prior information on the state evolution over time. Inspired by Variable Structure Multiple Model Estimator in [23], we propose CDKM to capture the context-dependent characteristics of occupants’ motion in the indoor space.
- $O_{k}\left(z_{k},y_{k}\right)=p\left(y_{k}|z_{k}\right)$: observation model, or how the unknown states and sensor observations relate. We will introduce PSMM where the relationship between locations and sensor observations is characterized by certain conditional probabilities and multiple sensor observations are combined via Bayes’ theorem.
- $C_{k}\left(z_{k},m_{k}\right)$: characteristic function that checks the validity of the correspondence between $z_{k}$ and $m_{k}$ using the contextual floormap.

Note that the prior knowledge abstracted from the floormap is inherently accommodated to this problem by defining characteristic function and parameterizing the transition model as will be elaborated in the following section.

### 3.2. Context-Dependent Kinematic Model

We assume that given $z_{k-1}$, $m_{k-1}$ and $m_{k}$, the current position/velocity $z_{k}$ follows a Gaussian distribution, of which the mean and covariance matrix are specified as

(2) $$p\left(z_{k}|z_{k-1},m_{k},m_{k-1}\right)∼N\left(F\left(m_{k-1},m_{k}\right)z_{k-1},GQ\left(m_{k-1},m_{k}\right)G^{\prime }\right)$$

The equivalent state space model of Equation (2) is given by

(3) $$\begin{array}{cc} & z_{k}=F\left(m_{k-1},m_{k}\right)z_{k-1}+Gv\left(m_{k-1},m_{k}\right)\\ & v\left(m_{k-1},m_{k}\right)∼N\left(0,Q\left(m_{k-1},m_{k}\right)\right) \end{array}$$

where $F\left(m_{k-1},m_{k}\right)\in R^{4\times 4}$ determines the mean of the distribution of the next state. Let *a* denote the acceleration, we have the following kinematic equations,

(4) $$\begin{array}{cc} x_{k} & =x_{k-1}+{\dot{x}}_{k-1}T+\frac{1}{2}aT^{2} \end{array}$$

(5) $$\begin{array}{cc} {\dot{x}}_{k} & ={\dot{x}}_{k-1}+aT \end{array}$$

where *T* is the sampling period. We will assume constant velocity in this paper, and model *a* as a Gaussian noise term. If we manipulate Equations (4) and (5) into matrix forms, then it can be identified that $F(m_{k-1},m_{k})$ has two possible values corresponding to moving or remaining static,

(6) $$F_{0}=\left[\begin{array}{cccc} 1 & 0 & T & 0\\ 0 & 1 & 0 & T\\ 0 & 0 & 1 & 0\\ 0 & 0 & 0 & 1 \end{array}\right],\;F_{1}=\left[\begin{array}{cccc} 1 & 0 & 0 & 0\\ 0 & 1 & 0 & 0\\ 0 & 0 & 0 & 0\\ 0 & 0 & 0 & 0 \end{array}\right]$$

$F_{1}$ imposes the velocity component of the state $z_{k}$ to be zero and $F=F_{1}$ when the context remains to be static space, *i.e.*, $m_{k-1}=m_{k}=SS$; otherwise, $F=F_{0}$.

The matrix *G* is given by

(7) $$G=\left[\begin{array}{cc} T^{2}/2 & 0\\ 0 & T^{2}/2\\ T & 0\\ 0 & T \end{array}\right]$$

$Q(m_{k-1},m_{k})$ stands for the process noise and, as the notation indicates, it is also a function of the context transition from k−1 to *k*. We will adopt the concept of directional noise to handle the constraints imposed by the contextual map. To see this, note that occupants in the free space ($m_{k-1}=m_{k}=FS$) can move in any direction with equal probability, therefore using equal process noise variance in both *x* and *y* direction, *i.e.*,

(8) $$Q_{0}=\left[\begin{array}{cc} \sigma_{f}^{2} & 0\\ 0 & \sigma_{f}^{2} \end{array}\right]\;$$

For occupants moving on the constrained space ($m_{k-1}=m_{k}=CS_{i},\forall i=1,\cdots ,R$) such as corridors, more uncertainty exists along than orthogonal to the corridor. Denote the variances along and orthogonal to the corridor by $\sigma_{a}^{2}$ and $\sigma_{o}^{2}$ ($\sigma_{a}^{2}>\sigma_{o}^{2}$), respectively, and the canonical direction of the constrained space $CS_{i}$ is specified by the angle $\phi_{i}$ (measured clockwise from y-axis). Then the process noise covariance matrix corresponding to the motion in the constrained space is given by

(9) $$\begin{array}{c} Q_{i}=\left[\begin{array}{cc} -\cos\phi_{i} & \sin\phi_{i}\\ \sin\phi_{i} & \cos\phi_{i} \end{array}\right]\left[\begin{array}{cc} \sigma_{o}^{2} & 0\\ 0 & \sigma_{a}^{2} \end{array}\right]\left[\begin{array}{cc} -\cos\phi_{i} & \sin\phi_{i}\\ \sin\phi_{i} & \cos\phi_{i} \end{array}\right] \end{array}$$

The preceding model specification incorporates the scenarios where the context remains the same during the time interval [k−1,k] and the occupant will keep the motion type defined by the two identical contexts. On the contrary, if the context switches during the time interval [k−1,k], we will assume a free motion pattern, *i.e.*, $F=F_{0}$, $Q=Q_{0}$. Table 2 summarizes our model given all possible context transitions.

### 3.3. Probabilistic Sensor Measurement Model

We construct probabilistic models for each sensor and multisensor fusion can be performed via Bayes’ rule. Assuming that $N_{s}$ different sensors function independently, then the observation model $p(y_{k}|z_{k})$ can be factored as

(10) $$\begin{array}{c} p\left(y_{k}|z_{k}\right)=\prod_{n=1}^{N_{s}}p\left(y_{k}^{n}|z_{k}\right) \end{array}$$

This actually forms a convenient and unified interface to combine distinctive sensor data by projecting the heterogeneous measurements ($y^{n}$) to the probability space via *likelihood function*, $p(y^{n}|z)$. If one more sensor is added into the system, then the observation model can be simply updated by multiplying the corresponding likelihood. Different likelihood functions requires being trained for different types of sensors.

**WiFi Measurement.** In the free space, the WiFi signal strength is a log linear function of the distance between the transmitter and receiver. However, due to the multipath effect caused by obstacles and moving objects in the indoor environments, the log linear relationship no longer holds. Previous work has proposed to adding a Gaussian noise term to account for the variations arising from the multipath effect; however, the simple model-based method can hardly guarantee a reasonable performance in practice. Another popular way is to construct a WiFi database comprising WiFi measurements at known locations to fingerprint the space of interest, but it requires onerous calibration to ensure the accuracy. We propose a novel WiFi modeling method based on a relatively small WiFi training set to accommodate for the complex variations of WiFi signals in the indoor space. The key insight is to use Gaussian process (GP) to model the WiFi signal where the simple model-based method provides a prior over the function space of GP.

We collect WiFi signal strength data at $N_{w}$ reference points over the space and let ${\left\{l^{j},y_{w}^{j}\right\}}_{j=1}^{N_{w}}$ denote the training dataset, where $l^{j}$ is a vector containing the distances of *j*th reference point to each of the WiFi APs deployed in the field and $y_{w}^{j}$ is the observed WiFi signal strengths. Assume the WiFi observations are drawn from the GP,

(11) $$\begin{array}{c} y_{w}∼\mathrm{GP}\left(\mu \left(l\right),k\left(l,l^{\prime }\right)\right) \end{array}$$

where the mean function μ(·) is imposed to be a linear model with the parameters adapted to the training samples. The covariance function k(·,·) takes the squared exponential form,

(12) $$\begin{array}{c} k\left(l,l^{\prime }\right)=\sigma_{f}^{2}\exp\left(-\frac{1}{2r^{2}}{\left(l-l^{\prime }\right)}^{2}\right)+\sigma_{n}^{2} \end{array}$$

where $\sigma_{n}^{2}$ stands for the variance of the additive Gaussian noise term in the observation process, and $\sigma_{f}^{2}$ and *r* are the hyperparameters of the GP. These parameters can be tweaked according to the training data, and we set $\sigma_{n}=4$, $\sigma_{f}=2$, r=5 in our experiments. At an arbitrary point $l_{*}$ in the space of interest, the posterior mean and variance of the WiFi signal $y_{*}$ are

(13) $$\begin{array}{cc} {\bar{y}}_{*} & =\mu \left(l_{*}\right)+K\left(l_{*},L\right){\left[K\left(L,L\right)+\sigma_{n}^{2}I\right]}^{-1}y_{w} \end{array}$$

(14) $$\begin{array}{cc} cov(y_{*}) & =K\left(l_{*},l_{*}\right)-K\left(l_{*},L\right){\left[K\left(L,L\right)+\sigma_{n}^{2}I\right]}^{-1}K\left(L,l_{*}\right) \end{array}$$

where L and $y_{w}$ are the vectors concatenated by ${\left\{l^{j}\right\}}_{j=1}^{N_{w}}$ and ${\left\{y_{w}^{j}\right\}}_{j=1}^{N_{w}}$, respectively. $K(l_{*},L)$ denotes the $1\times N_{w}$ matrix of the covariances evaluated at all pairs of training and testing points, and similarly for the other entries K(L,L) and $K(L,l_{*})$. In previous work using GP to model the WiFi signal strength [24], the WiFi signal is assumed to follow the Gaussian distribution with the mean and variance given by Equations (13) and (14), respectively. However, the posterior variance derived from GP is a indicator of estimation confidence. It depends largely on the density of training samples in the vicinity of the evaluated position. That is, if the evaluated point $l_{*}$ happens to fall into the area that is densely calibrated, then the posterior variance will be relatively small. The posterior variance derived from GP cannot truly reflect the variations of WiFi signals over time. Therefore, instead of using the posterior Variance (14) in classical predictive equations, we model the likelihood as

(15) $$y_{*}∼N\left({\bar{y}}_{*},\sigma_{n}^{2}\right)$$

**Ultrasonic Measurement.** Essentially, each of the ultrasonic sensors in the ultrasonic station can output the distance to the occupant passing in front of it. However, due to the missing data and measurement noise, the distance measurement is not always steady. Here, we will consider the ultrasonic station to be a binary sensor to indicate the occupancy in its detection zone. To be specific, the likelihood function is modeled as

(16) $$\begin{array}{c} p(y_{k}<\eta |z_{k}\;\text{in}\;\text{the}\;\text{detection}\;\text{zone})=1 \end{array}$$

where *η* is the threshold for ultrasonic measurements.

### 3.4. Characteristic Function

The characteristic function imposes constraints on the correspondence between the position and the context, and embodies the prior knowledge available from the floormap. In the preceding section, we have defined a function M that sets up the relationship between the context and the position/velocity, *i.e.*, $m_{k}=M\left(z_{k}\right)$, and M can be readily read out from the contextual map. We thereby define the characteristic function to be

(17) $$C_{k}\left(z_{k},m_{k}\right)=I\left[M\left(z_{k}\right)-m_{k}=0\right]$$

where I[·] is an indicator function. In other words, the characteristic function enforces the local correspondence defined by M.

## 4. Context-Augmented Particle Filter

In this section, we will discuss how to perform inference on the underlying factor graph of the tracking problem we formulated previously. The particle filter is a technique for implementing a recursive Bayesian filter by Monte-Carlo simulations [25]. The key idea of particle filter is to represent the required posterior density function by a set of random samples or “particles” associated with discrete probability mass, and compute the state estimate based on these “particles”. The original particle filter proposed by Gordon *et al*. [26] was designed for a simple hidden Markov chain, which is also a cycle-free factor graph, using the Sampling Importance Resampling (SIR) algorithm to propagate and update the particles. However, the factor graph in our problem, as illustrated in Section 4, does have cycles due to the introduction of the context variable, and only approximate inference algorithms exist. We present a recursive approximate inference method for the cyclic factor graph by extending the particle filter and the resulting algorithm is termed *Context-Augmented Particle Filter* (CAPF).

To see the operation of the CAPF, consider a set of particles ${\left\{z_{k-1}^{i},m_{k-1}^{i}\right\}}_{i=1}^{N}$ that represents the posterior distribution $p(z_{k-1},m_{k-1}|y_{1:k-1})$ of the state. Note that $m_{k-1}^{i}$ can be uniquely determined by $z_{k-1}^{i}$ via the characteristic function. At time *k*, we have some new measurement $y_{k}$. It is required to construct a new set of particles ${\left\{z_{k}^{i},m_{k}^{i}\right\}}_{i=1}^{N}$ which characterizes the posterior distribution $p(z_{k},m_{k}|y_{1:k})$. Now, suppose we have an “*oracle*" that is capable of providing the context value $m_{k}^{i}$ of the corresponding $z_{k}^{i}$ even before we generate $z_{k}^{i}$’s, then our task is equivalent to draw samples from the distribution

(18) $$\begin{array}{c} p(z_{k}|m_{k},y_{1:k}) \end{array}$$

This can be carried out in two steps: First, the historical density $p(z_{k-1},m_{k-1}|y_{1:k-1})$ is propagated via the transition model $p(z_{k}|z_{k-1},m_{k},m_{k-1})$ to produce the prediction density

(19) $$\begin{array}{cc} p(z_{k}|m_{k},y_{1:k-1}) & =\int p\left(z_{k}|z_{k-1},m_{k}\right)p\left(z_{k-1}|y_{1:k-1}\right)dz_{k-1} \end{array}$$

where $p\left(z_{k}|z_{k-1},m_{k}\right)=p\left(z_{k}|z_{k-1},m_{k},m_{k-1}\right)$ since $m_{k-1}$ is completely determined conditioning on $z_{k-1}$. Second, our interested density $p(z_{k}|m_{k},y_{1:k})$ can be updated from the prediction density using Bayes’ theorem,

(20) $$\begin{array}{cc} p(z_{k}|m_{k},y_{1:k}) & =\frac{p\left(y_{k}|z_{k}\right)p\left(z_{k}|m_{k},y_{1:k-1}\right)}{p(y_{k}|y_{1:k-1},m_{k})} \end{array}$$

(21) $$\begin{array}{cc} & =\gamma p\left(y_{k}|z_{k}\right)p\left(z_{k}|m_{k},y_{1:k-1}\right) \end{array}$$

where *γ* is a normalization constant. Thus, Equation (19) and Equation (20) form a recursive solution to Equation (18). In particle filter framework, the aforementioned prediction and update steps are performed by propagating and weighting the random samples.

**Prediction Step.** In the prediction phase, we generate the predicted particles by

(22) $$\begin{array}{c} {\tilde{z}}_{k}^{i}∼p\left(z_{k}|z_{k-1}^{i},{\tilde{m}}_{k}^{i},m_{k-1}^{i}\right) \end{array}$$

where ${\left\{{\tilde{m}}_{k}^{i}\right\}}_{i=1}^{N}$ is a set of particles representing the estimates of $m_{k}$ produced by the “oracle”. Given the different possible values of $m_{k-1}^{i}$ and ${\tilde{m}}_{k}^{i}$, ${\tilde{z}}_{k}^{i}$ will be sampled from different models, detailed in Table 2. We will then perform sanity check on newly generated particles, where the particles ${\tilde{z}}_{k}^{i}$ absent from the reachable set of $z_{k-1}^{i}$ will be eliminated.

**Update Step.** To update, each predicted particle ${\tilde{z}}_{k}^{i}$ is assigned with a weight proportional to its likelihood.

(23) $$\begin{array}{c} {\tilde{w}}_{k}^{i}=p\left(y_{k}|{\tilde{z}}_{k}^{i}\right) \end{array}$$

The weight is then normalized by

(24) $$\begin{array}{c} w_{k}^{i}=\frac{{\tilde{w}}_{k}^{i}}{\sum_{i=1}^{N}{\tilde{w}}_{k}^{i}} \end{array}$$

We resample *N* times with replacement from the set ${\left\{{\tilde{z}}_{k}^{i}\right\}}_{i=1}^{N}$ using weights ${\left\{w_{k}^{i}\right\}}_{i=1}^{N}$ to obtain a new set of samples ${\left\{z_{k}^{i}\right\}}_{i=1}^{N}$ such that $p\left(z_{k}^{i}={\tilde{z}}_{k}^{i}\right)=w_{k}^{i}$. Correspondingly, the contexts $m_{k}^{i}$’s are obtained through the characteristic function, *i.e.*,

(25) $$\begin{array}{c} m_{k}^{i}=M\left(z_{k}^{i}\right) \end{array}$$

**“Oracle” Design.** The oracle is supposed to be able to answer the query about the next possible contexts $m_{k}$, based upon which the position/velocity component of the state can be properly propagated according to different transition models. For computational efficiency, we adopt a simple discriminative model to produce ${\tilde{m}}_{k}$’s. Given a small database of WiFi fingerprints, we apply the K-Nearest Neighbors (K-NN) algorithm and a modified distance weighted rule to generate an empirical distribution of the context. To be specific, let the WiFi database be denoted by ${\left\{\left(m^{j},y_{w}^{j}\right)\right\}}_{j=1}^{N_{w}}$, and $N_{w}$ is the number of WiFi fingerprints. When the new WiFi observation $y_{k}$ is querying the possible contexts, the *K* nearest neighbors of $y_{k}$ are found among the given training set. Let these *K* nearest neighbors of $y_{k}$, with their associated context, be given by ${\left\{\left(m^{j^{\prime }},y_{w}^{j^{\prime }}\right)\right\}}_{j^{\prime }=1}^{K}$. In addition, let the corresponding distances of these neighbors from $y_{k}$ be given by $d^{j^{\prime }}$, $j^{\prime }=1,\cdots ,K$. The weight attributed to the $j^{\prime }$th nearest neighbor is then defined as

(26) $$\begin{array}{c} {\tilde{q}}^{j^{\prime }}=\frac{d^{K}-d^{j^{\prime }}}{d^{K}-d^{1}},\;j^{\prime }=1,\cdots ,K \end{array}$$

We then normalize the weights, $q^{j^{\prime }}=\frac{{\tilde{q}}^{j^{\prime }}}{\sum_{j^{\prime }=1}^{K}{\tilde{q}}^{j^{\prime }}}$, and sample the context according to the following discrete probability distribution,

(27) $$\begin{array}{c} P\left({\tilde{m}}_{k}=m^{j^{\prime }}\right)=\left\{\begin{array}{cc} q^{j^{\prime }}\left(1-\alpha \right)+\alpha , & m^{j^{\prime }}=m_{k-1}\\ q^{j^{\prime }}\left(1-\alpha \right), & m^{j^{\prime }}\neq m_{k-1} \end{array}\right. \end{array}$$

where *α* is a context resilience factor and α∈[0,1]. We incorporate *α* to accommodate for the prior knowledge that the context will not change too often and to make the “oracle” more robust to the observation noise. Moreover, for the particles on the boundary of distinctive contexts, ${\tilde{m}}_{k}$ is equally probable to be these contexts. The pseudo-code of the CAPF algorithm is provided in Algorithm 1.

**Algorithm 1** Context-Augmented Particle Filter **function**
CAPF($y_{1:T},wifi\_database,reachable\_set$)   Initialization:   Uniformly generate *N* samples ${\left\{z_{0}^{i}\right\}}_{i=1}^{N}$   Set $m_{0}^{i}=M\left(z_{0}^{i}\right)$, $w_{0}^{i}=N^{-1}$, i=1,⋯,N   **for**
k=1,⋯,T
**do**    **for**
i=1:N
**do**    Context Estimate:    **if**
$z_{k-1}^{i}$ on the boundary of ${\left\{m_{b}\right\}}_{b=1}^{B}$
**then**     Uniformly sample ${\tilde{m}}_{k}^{i}$ from ${\left\{m_{b}\right\}}_{b=1}^{B}$    **else**     Sample ${\tilde{m}}_{k}^{i}$ from Equation (27)    **end if**    Prediction Step:    ${\tilde{z}}_{k}^{i}∼p\left(z_{k}|z_{k-1}^{i},{\tilde{m}}_{k}^{i},m_{k-1}^{i}\right)$    Discard particles ${\tilde{z}}_{k}^{i}\notin reachable\_set\left(z_{k-1}^{i}\right)$    Update Step:    Compute weight ${\tilde{w}}_{k}^{i}=p\left(y_{k}|{\tilde{z}}_{k}^{i}\right)$   **end for**   Normalize weights: $w_{k}^{i}=\frac{{\tilde{w}}_{k}^{i}}{\sum_{i=1}^{N}{\tilde{w}}_{k}^{i}}$   Resampling:   Select *N* particle indices $i^{\prime }\in \left\{1,\cdots ,N\right\}$ according to weights ${\left\{w_{k}^{i}\right\}}_{i=1}^{N}$   Set $z_{k}^{i}={\tilde{z}}_{k}^{i^{\prime }}$ and $w_{k}^{i}=N^{-1}$   Set $m_{k}^{i}=M\left(z_{k}^{i}\right)$   Estimate:   ${\hat{z}}_{k}=\sum_{i=1}^{N}w_{k}^{i}z_{k}^{i}$  **end for**  **return**
${\hat{z}}_{1:T}$ **end function**

## 5. Performance Evaluation

**Figure 5:**
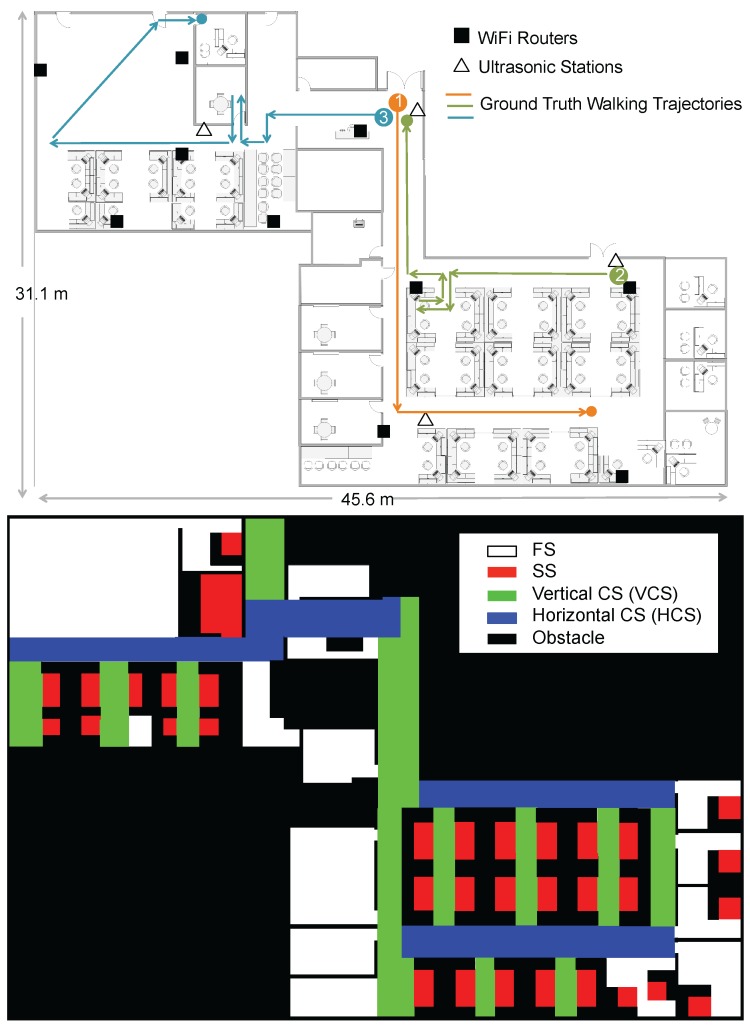
The floormap (**top**) and corresponding contextual map (**bottom**) of the testbed. Four different contexts (FS, SS, VCS, HCS) are defined and color coded as illustrated in the legend.

Our experiment was carried out in the Singapore–Berkeley Building Efficiency and Sustainability in the Tropics (SinBerBEST) located in CREATE Tower at the National University of Singapore campus, which is a typical office environment consisting of cubicles, individual offices, corridors and obstacles like walls, desks, *etc*. The total area of the testbed is around 1000 m${}^{2}$. There are 10 WiFi routers and four ultrasonic stations deployed in the testbed in total. We utilize TP-LINK TL-WDR4300 Wireless N750 Dual Band Routers (manufactured in Shenzhen, China) as WiFi APs and HC-SR04 Ultrasonic Sensors (manufactured in Shenzhen, China) as the components of ultrasonic stations. The floormap and the corresponding contextual map are shown in Figure 5. Different contexts are colored differently in the contextual map. The static space contains the seating areas in the cubicles and offices, where occupants hardly move. The corridors of horizontal and vertical directions are considered to be two types of constrained spaces (HCS and VCS, respectively). The free space includes the open areas where occupants can freely move. We seek to answer the questions including how well MapSentinel is able to track the occupant, and whether the map information exploited by way of MapSentinel can bring additional benefits to the tracking performance.

**Experimental methodology.** In a real-world setting, we expect the occupant to carry the smartphone as they walk through various sections of an indoor space. Moreover, occupants are unlikely to walk continuously; they would walk between locations of special interest and dwell at certain locations for a significant length of time. Our experiment aims at emulating these practical scenarios in an office environment and incorporating all the contexts defined in our model. Therefore, the following routes were designed as the ground truth for evaluation: (1) A enters the office from the front gate and walks through the corridors to find her colleague (different CSs are included); (2) B enters the office from the side door, walks to her own seat, stays there for a while and exits the office from the front gate (CSs, SS are included); (3) C enters the office from the front gate, walks through corridors, takes some time at her office and goes to the open area (CSs, SS, FS are included). We asked the experimenter to behave as usual when walking in the space. At the same time, the WiFi APs and ultrasonic stations constantly collect the measurements and send them to the central server. To obtain the ground truth at the sampling time of the tracking system, we mark the ground with a 1 m grid on the pre-specified route and ask the experimenter to create lap times with a stopwatch when happening to be on the grid. By recording the starting time of the experiment, we can obtain the time stamp of each grid and then interpolate the ground truth at the sampling time.

**Figure 6:**
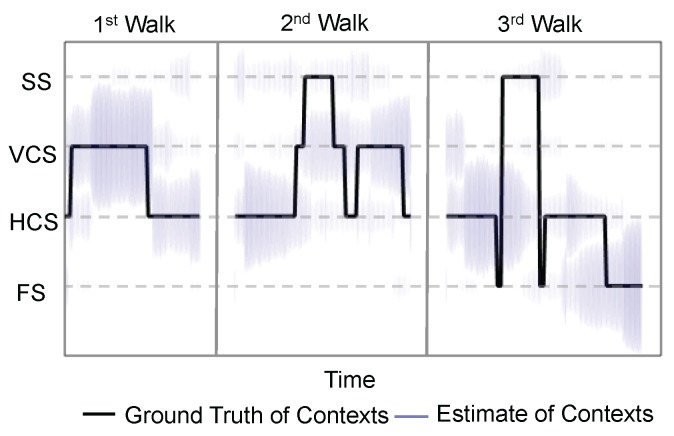
The context estimate produced by the “oracle" *versus* the ground truth context. The radius of the purple cloud is proportional to the number of particles of the estimated context which the cloud is centered around.

**Figure 7:**
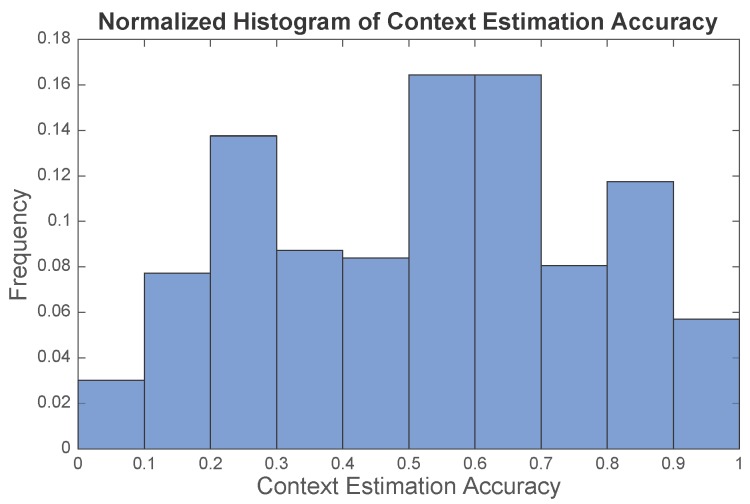
Normalized histogram of context estimation accuracy of the “oracle”. The mean accuracy is 52.41%.

**Does the “oracle” work?** The current context estimation done by the “oracle” is critical to the CAPF algorithm, as the tuple of the current and previous context jointly steer the states in our model. Here, we would like to evaluate the context prediction performance of the “oracle” we constructed in light of several design rules presented in the Section 4. Figure 6 illustrates the result of the context estimation for different walks. Since the context estimates are represented by a set of particles in the algorithm, we visualize the context estimate by the purple lines centered at the possible contexts, and the lengths of the purple lines are scaled by the proportions of the particles of different contexts. Ideally, the purple cloud should scatter around the ground truth context. Figure 6 suggests that the estimates given by the “oracle” can generally capture the ground truth. Evidently, the context estimate is not perfect, especially for the static space (SS). However, these approximate “ground truths” essentially present other possibilities of the current context and avoids particles trapping in the static space. We define the context estimation accuracy to be the ratio of the number of particles with correct context estimate to the total number of particles. The context estimation accuracy is calculated for each time step of the experiments, and the empirical distribution of the context estimation accuracy is illustrated in Figure 7, where the mean accuracy is 52.41%. With this noisy “oracle”, the system can achieve median tracking error of 1.96 m, while the tracking error would be 1.84 m if a perfect “oracle” was utilized. Therefore, our work has the potential to be further improved with a more advanced “oracle” design.

**Figure 8:**
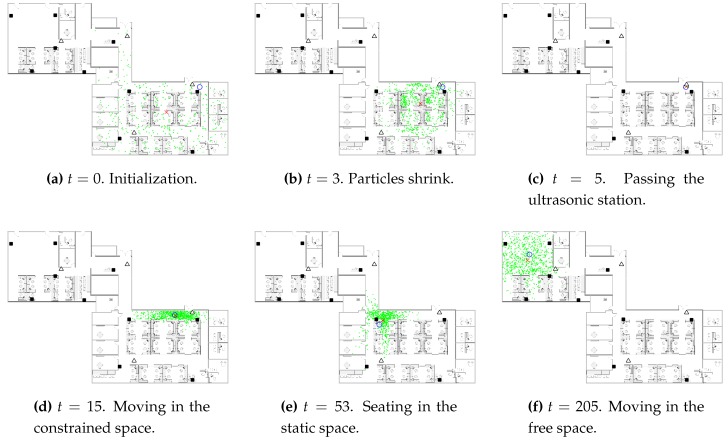
The snapshots of the intermediate steps of the CAPF algorithm visualized. The location estimate, ground truth location, particles are presented by the red cross, blue circle, green dots, respectively. As before, the black square and white triangles give the positions of WiFi routers and ultrasonic stations.

Figure 8 demonstrates some snapshots of the CAPF algorithm in progress. At the beginning, the particles are initialized to be uniformly distributed in the space. In addition, the spread of the particles shrinks as the new WiFi observations come. When the ultrasonic station reports a detection, the particles are concentrated in the corresponding detection zone. As the occupant exits the detection zone, the particles spread out along the direction of the corridor. When the occupant sits in the cubicle, the particles distribute over the seating area as well as some possible routes through which the occupant might leave the seating area. The particles distribute evenly along different directions when the occupant is moving in the free space, in which case our model is identical to the traditional constant velocity dynamic model for the particle filter.

**Figure 9:**
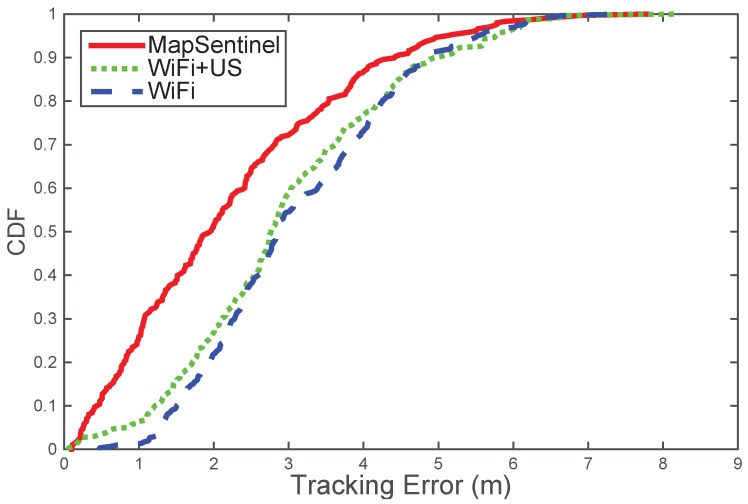
Tracking performance of MapSentinel, the fusion system of WiFi and ultrasound sensor, the pure WiFi system. The median tracking accuracy of the MapSentinel is 1.96 m, MapSentinel can achieve the performance improvement of 31.3% over the purely WiFi-based tracking system, 29.1% over the fusion system.

**Figure 10:**
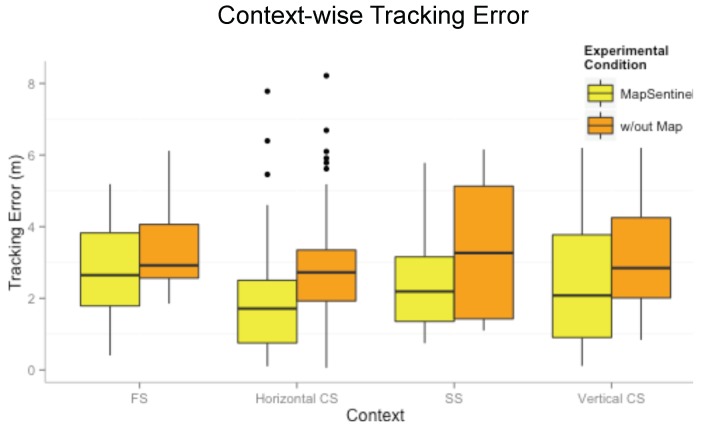
Tracking error in different contexts for the MapSentinel and the WiFi+Ultrasound system.

**MapSentinel’s tracking performance.** We aggregate the data from different walks and compare the performance of MapSentinel against the fusion system of WiFi and ultrasonic station without leveraging the floormap information, as well as the purely WiFi-based tracking system. The tracking error distributions are depicted in Figure 9. As can be seen, the MapSentinel achieves an essential performance improvement, 31.3% over the WiFi tracking system and 29.1% over the fusion scheme. Note that adding the ultrasonic calibration into the WiFi system is able to realize a small amount of accuracy increment. Due to the high degree of uncertainty of WiFi signals, the effect of ultrasonic calibration will not last for long. The map information elongates the effect of the ultrasonic calibration via imposing additional constraints to the motion, and that is why MapSentinel greatly enhances the tracking performance compared with the purely WiFi-based system. We also evaluate the tracking performance in different contexts, and the result is shown by boxplots in Figure 10. Here, “without map” means using the WiFi and ultrasonic sensing systems without taking into account the reachable set as well as the context-dependent kinematic model. A unified dynamical model, the free space model, is applied in this case, and a traditional particle filter is implemented to estimate the location. As can be readily read from the figure, the MapSentinel performs better in all contexts. More significant increase is achieved in constrained spaces and static spaces, as expected.

**Figure 11:**
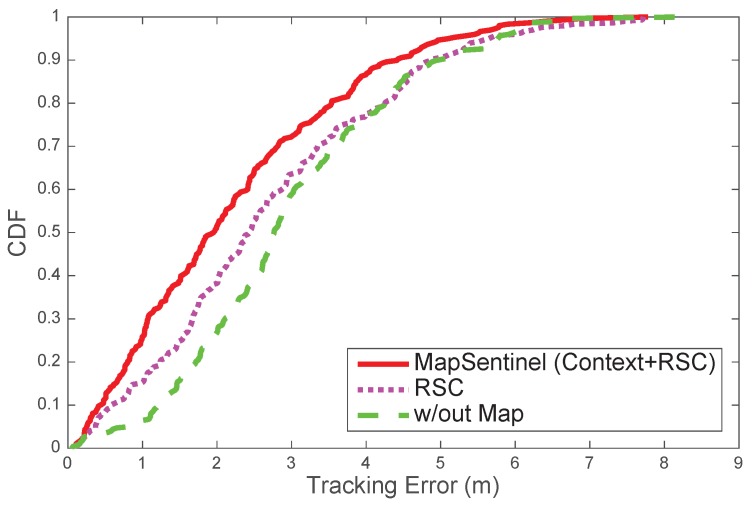
Tracking performance of different usage of floormap information. “RSC” stands for reachable set check. MapSentinel extracts the context information from the floormap, and simultaneously eliminates the particles falling outside the reachable set. MapSentinel is compared with the tracking system without using context information (*i.e*., only performing RSC) and the one without using the map information at all. The median tracking errors of MapSentinel, the system only performing RSC, and the one without exploiting the floormap information are 1.96 m, 2.44 m and 2.77 m, respectively.

Figure 11 compares the performance of tracking systems with distinctive floormap usage. MapSentinel exploits the floormap information in two folds: first, MapSentinel integrates the context information into the kinematic model, and the movement patterns of people on different locations of the map are better captured. Secondly, MapSentinel takes into account the speed restrictions as well as physical obstacles in the indoor space by checking if the particles fall inside the reachable set at each time step. The second fold of the floormap information has been widely utilized in the previous work, while the context information is less explored. We therefore compare the tracking error of our system with the one that merely uses the reachable conditions. Figure 11 shows that incorporating information about physical constraints, as the previous work did, is surely beneficial to the tracking system. Particularly, the performance can be further improved by 19.8% by introducing the context information into the tracking system.

**Figure 12:**
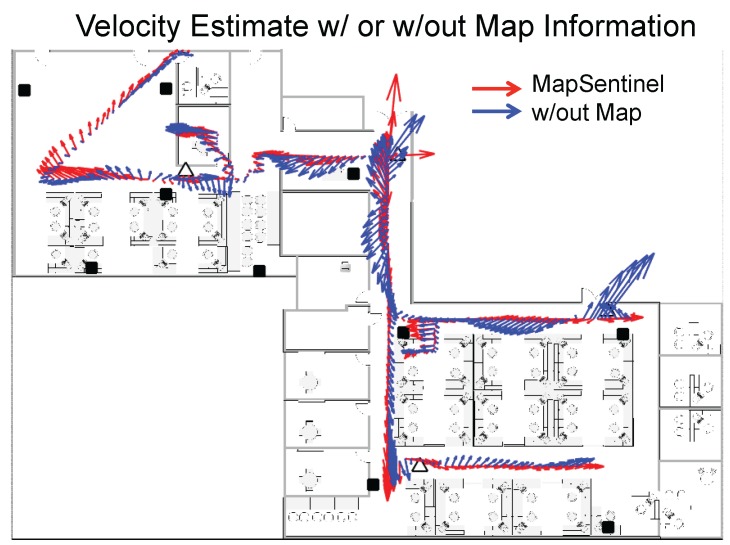
The velocity estimation for the MapSentinel and the WiFi+Ultrasound system. The vector indicates the speed and direction of the estimated motion.

To better understand how the map helps improve the location estimation, we demonstrate the velocity estimation of different tracking schemes in Figure 12. Typically, the occupants will not perform complex motions in the indoor space due to the constraints of the wall and other barricades. The more the velocity estimate deviates from the canonical directions defined by the indoor environment, the worse the tracking performance can be. Using the fusion schemes of WiFi and ultrasonic calibration, only the location is the observable state. The velocity estimates depend largely on the location estimate and it has little effect in smoothing out the location estimate. Hence, extensive research has been focusing on using inertial measurements to perform dead reckoning, which makes the velocity observable. Analogously, the MapSentinel creates a *virtual* inertial sensor for the occupant, which mimics the actual inertial sensor to provide the possible walking speed and directions. As is shown in Figure 12, the velocity estimation without map information tends to point to any direction while the MapSentinel constrains the velocity via the context-dependent kinematic model.

## 6. Conclusions

This paper presents MapSentinel, a system for real-time location tracking that emphasizes both non-intrusiveness and accuracy. The non-intrusive sensing networks comprise the modified WiFi access points and the ultrasonic calibration stations. The MapSentinel makes novel attempts to exploit the floormap information by categorizing the indoor space into different contexts to capture the diversity of typical motion characteristics. This mimics having an inertial sensor attached to the occupant to obtain the knowledge of velocity. We formalize the fusion of floormap information as well as the noisy sensor readings using the Factor Graph, and develop the Context-Augmented Particle Filtering algorithm to efficiently solve real-time walking trajectories. Our evaluation in the large typical office environment shows that MapSentinel can achieve the performance improvement of 31.3% over the purely WiFi-based tracking system. MapSentinel is among the early attempts to obviate the need for the inertial sensors in indoor tracking, and our results are promising.

For future work, we would like to explore multiple occupant tracking. The ultrasonic sensor is essentially anonymous and cannot identify the occupant entering its detection zone. The WiFi access points are able to identify the occupant from the MAC address of the mobile device and can approximately tell which occupant is approaching the ultrasonic station. The ultrasonic calibration will work if the occupant can be identified with the MAC information without ambiguity; however, if the identity of the occupant within the range cannot be uniquely determined, as in the crowded scenario, the calibration may not work effectively. Further work to reliably track multiple occupants is necessary. Moreover, we would like to integrate our tracking method to the control of lighting and ventilation systems to improve energy efficiency of buildings.

## Figures and Tables

**Table 1:** Components of contextual floormap.

Context	Symbols	Motion Characteristics
Free Space	FS	Move freely, e.g., rooms
Constrained Space	CS	Move along canonical direction, e.g., corridors
Static Space	SS	Stay static, e.g., cubicles

**Table 2:** Context-dependent kinematic models.

Context Transition	Model Specification	
	$F(m_{k-1},m_{k})$	$Q(m_{k-1},m_{k})$
$m_{k-1}=m_{k}=FS$	$F_{0}$	$Q_{0}$
$m_{k-1}=m_{k}=CS_{i}$	$F_{0}$	$Q_{i}$
$m_{k-1}=m_{k}=SS$	$F_{1}$	$Q_{0}$
$m_{k-1}\neq m_{k}$	$F_{0}$	$Q_{0}$
